# Predictive factors affecting stress among nurses providing care at COVID‐19 isolation hospitals at Egypt

**DOI:** 10.1002/nop2.652

**Published:** 2020-10-11

**Authors:** Abdelaziz Hendy, Ahmed Abozeid, Gehan Sallam, Hadya Abboud Abdel Fattah, Fadia Ahmed Abdelkader Reshia

**Affiliations:** ^1^ Assistant Lecturer at Pediatric Nursing Faculty of Nursing Ain Shams University Cairo Egypt; ^2^ Assistant Lecturer at Medical Surgical Nursing Faculty of Nursing Ain Shams University Cairo Egypt; ^3^ Clinical Research Nurse at Obstetrics and Gynecology Department UAE University College of Medicine and Health Sciences Al Ain United Arab Emirates; ^4^ Senior Lecturer at Fatima College of Health Sciences Al Ain United Arab Emirates; ^5^ Assistant professor at Department of Nursing College of Applied Medical Sciences Jouf University Sakākā Saudi Arabia; ^6^ Lecturer at Critical Care and Emergency Nursing Faculty of Nursing Mansoura University Mansoura Egypt

**Keywords:** COVID‐19, isolation, nurses, stress

## Abstract

**Aims:**

To examine predictive factors affecting stress among nurses providing care at COVID‐19 Isolation Hospitals at Egypt.

**Methods:**

A cross‐sectional study conducted in five Isolation governmental hospitals for COVID‐19. 374 nurses included at the study. Characteristic forms, factors affecting nurses’ stress and Nursing Stress Scale (NSS) were used to collect data.

**Results:**

(52.1%) of studied nurses had moderate level of total nursing stress scale. Also, (26.2%) of them had severe level, while (13.4% & 8.3%) of them had mild and normal level, respectively. Mean *SD* score of studied nurses regarding to total nursing stress scale was 99.47 ± 10.671.

**Conclusions:**

Training for COVID‐19, availability of PPE, educational level and attention of hospital administration were negative predictor factors for nurses’ stress, while having children, people showed that COVID‐19 is stigma, fears of infection, workplace, fear of transmission infection for family and nurse to patient ratio were positive predictors.

## 1 INTRODUCTION

Coronavirus disease‐2019 (COVID‐19) is an infectious disease caused by severe acute respiratory syndrome coronavirus 2 (SARS‐CoV‐2). The disease firstly diagnosed at December 2019 in Wuhan, which capital of China country, and has since spread globally and caused the ongoing 2019–20 coronavirus pandemic (COVID & Team, 2020). Communal symptoms as fever, dry cough or sputum production, fatigue, loss of smell and shortness of breath. While most cases result in mild symptoms, some progress to viral pneumonia and multi‐organ failure. Emergency symptoms include persistent chest pain or pressure, difficulty breathing, confusion and bluish face or lips (Rothan & Byrareddy, 2020).

The time from exposure to onset of symptoms is classically around five days but may range from two days to two weeks. As last second week of May 2020, more than 4.35 million cases have been reported across 185 countries and territories, more than 1.55 million people have recovered and more than 297,000 deaths (World Health Organization [WHO], 2020). As of the evening of May 13, there were 10.431 affirmed cases of COVID‐19 and 556 deaths in Egypt. The 2020 coronavirus widespread in Egypt is a portion of a continuous around the world coronavirus widespread. The primary cause of COVID‐19 in Egypt was affirmed on 14 February 2020 (Ministry of Health and Population Egypt [MOHP], 2020).

Throughout the COVID‐19 pandemic, nurses are experiencing fear, pressure, tiredness, ongoing emotional trauma and isolation (Nobles et al., 2020). This ongoing trauma and stress impact nurses’ mental health, feeling safe and providing the finest possible care (Cheung et al., 2020).

Previous studies have shown that during sudden natural disasters and infectious diseases, nurses will sacrifice their own needs to actively participate in the anti‐epidemic work and make selfless contributions out of moral and professional responsibility (Maben & Bridges, 2020). At the same time, nurses would be in a state of physical and mental stress and feel isolated and helpless in the face of health threats and pressure from the high‐intensity work caused by such public health emergencies (Aliakbari et al., 2015).

Stress is an imprecise term, which is usually defined in terms of the internal and external stressful conditions (Horowitz & McIntosh, 2017) & (Zhou et al., 2020). Stress can have a significant impact on individual nurses, and their ability to accomplish tasks and more specifically, poor decision‐making, lack of concentration, apathy, decreased motivation and anxiety may impair job performance creating uncharacteristic errors (Jun et al., 2020).

## 2 MATERIALS AND METHODS

### 2.1 Aims of study

The purpose of this study was to examine the predictive factors affecting stress among nurses providing care at COVID‐19 isolated hospitals in Egypt.

### 2.2 Method study design and procedure

A cross‐sectional study was used to examine the predictors’ factors affecting stress among nurses providing care at COVID‐19 isolated hospitals in Egypt. The study was carried out at five isolated governmental hospital for COVID‐19 (Ahmed Maher Teaching Hospital, lmpapa general Hospital, New Qalioub Hospital and Banha Fever Hospital in Qalioubia, 6 October Insurance Hospital), and these hospitals include intensive care units, medical rooms and well equipped with ventilators, cardiac monitor, defibrillators and cardioversion, crash carts, all personal protective equipment (PPE). Symptomatic patients admitted firstly to Triage Hospitals which was identified by the Ministry of Health, then swabs done for them and they transfer the patients to isolated hospital after the positive swabs are confirmed and the Ministry of Health informs the patient with the closest hospital to his residence.

The study subjects were selected among nurses providing care for patient at COVID‐19 quarantine regardless of their age, gender, qualifications and experience. 524 was the total number of nurses in the five selected hospitals, 374 nurses were selected using the convenience sampling method. All participants are asked to fill an electronic questionnaire sent to their mobiles for commitment to social spacing and after spending a period of 14 days in COVID‐19 quarantine hospitals and also after explaining the purpose of the study and confirming the privacy of the data.

### 2.3 The instruments

One tool was developed and used to collect data after reviewing related literatures (Mo et al., 2020; Pappa, 2020) and translated into Arabic Language, reliability and validity were done and it included three parts as following:

First Part: It contains demographic data of nurses as age, gender, marital status, educational level, nursing experience, workplace and presence of children.

Second Part: It included factors affecting nurses’ stress level which consisted of 9 items as follows: participated in quarantine for infectious disease previously, provide a training courses related to COVID‐19, history of psychiatric illness, fears of infection…. etc.

Third Part: The Nursing Stress Scale (NSS) that adopted from Gray‐Toft & Anderson, 1981), the scale consists of 34 items: these items were distributed into seven heterogeneous and potentially stressful situations, including death and dying patients (7 items), conflict with physicians (5 items), inadequate preparation (3 items), lack of staff support (3 items), conflict with other nurses (5 items), workload (6 items) and uncertainty concerning treatment (5 items). A 4‐point Likert scale was used to indicate the frequency of work stressors experienced by nurses from never (1), occasionally (2), frequently (3), very frequently (4).

A higher score indicates a higher frequency of work stressors experienced by the participants. Internal consistency of the NSS was measured by the Spearman–Brown coefficient (79), the Guttman split‐half coefficient (79), and a coefficient alpha (89) (Gray‐Toft & Anderson, 1981), the scale scores ranging from 34–136.

### 2.4 Content validity and reliability

It was ascertained by a group of experts in critical and psychiatric nursing department (5). Their opinions elicited regarding the format, layout, consistency, accuracy and relevancy of the tools. Reliability the pretest was carried out to test the reliability Cronbach's Alpha for questionnaire = 0.864.

### 2.5 Ethical considerations

Each nurse was informed about the purpose and benefits of the study in the first part before participation at the study, where everyone cannot be starting the questionnaire without consent to participate in data collection in the current study. The nurses were assured that all data were used for research purpose only and each one was informed of the rights to refuse participation in the study or withdraw at any time before completing the questionnaire with no consequences.

### 2.6 Data collection

The researchers collected the data from the nurses after spending a period of 14 days in COVID‐19 quarantine hospitals by sending the questionnaire after translating it into Arabic by email and social media after sending a message explaining the purpose of the study and confirming the privacy of the data.

### 2.7 Statistical analysis

Computerized data entry and Statistical analysis were fulfilled using the Statistical Package for Social Sciences (SPSS) version 22. Linear regression model is a linear approach to modelling the relationship between a scalar response and one or more explanatory variables. Chi‐square statistic is commonly used for testing relationships between categorical variables.

## 3 RESULTS

### 3.1 Demographic data

Table 1 presented that, 50% of the studied nurses were aged between 20–<30 years, the mean age of them 32.06 (*SD* 3.90) years. As regard to gender and marital status, 67.4% and 59.1% of the studied nurses were female and not married, respectively. Also, 55.6% of them did not have children. Moreover, 70.6% of the studied nurses working at ward units. In relation to the educational level of nurses under study, it was found that 54.8% of them had Technical Institute of Nursing. Also, 52.4% of the studied nurses their years of experience were < 10 years, with mean 12.87 (*SD* 5.08) years (Table (1) the studied nurses’ distribution according to their characteristics (*n* = 374).

**TABLE 1:** The studied nurses’ distribution according to their characteristics (*n* = 374)

Items	*N*	%
Age (year)		
20 to <30	187	50
30 to <40	110	29.4
40–50	77	20.6
29.4	32.06 ± 3.90	
Gender		
Male	122	32.6
Female	252	67.4
Marital status		
Married	153	40.9
Not Married	221	59.1
Do you have children		
Yes	166	44.4
No	208	55.6
Work place		
Critical care units	110	29.4
Ward units	264	70.6
Educational level		
Postgraduate	18	4.8
Bachelor nursing degree	125	33.4
Technical Institute of Nursing	205	54.8
Diploma nursing degree	26	7
Years of experience		
<10	196	52.4
10 to <20	114	30.5
≥20	64	17.1
Mean *SD*	12.87 ± 5.08	

### 3.2 Nursing stress Scale (NSS)

Table 2 showed that the mean *SD* score of studied nurses regarding to Workload and Inadequate Preparation was 21.84 (*SD* 5.367) and 9.92 (*SD* 2.436), respectively. Also, the mean score of them regarding to Lack of Staff Support, Death and dying and Uncertainty concerning treatment was 9.41 (*SD* 3.491), 21.37 (*SD* 5.247) and 14.51 (*SD* 4.148), respectively. Moreover, the mean *SD* score of studied nurses regarding to Conflict with Physicians and Conflict with nurses was 11.96 (*SD* 6.372) and 11.04 (*SD* 5.239), respectively. Finally, the mean *SD* score of studied nurses regarding to total nursing stress scale was 99.47 (*SD* 10.671) (Table (2): Most stressful subscales and least stressful subscales perceived by nurses. (*n* = 374).

**TABLE 2:** Most stressful subscales and least stressful subscales perceived by nurses. (*n* = 374)

Subscales	Items	Mean	*SD*
Workload	6	21.84	5.367
Inadequate preparation	3	9.92	2.436
Lack of staff support	3	9.41	3.491
Death and dying	7	21.37	5.247
Uncertainty concerning treatment	5	14.51	4.148
Conflict with physicians	5	11.96	6.372
Conflict with other nurses	5	11.04	5.239
Total	34	99.47	10.671

**FIGURE 1:**
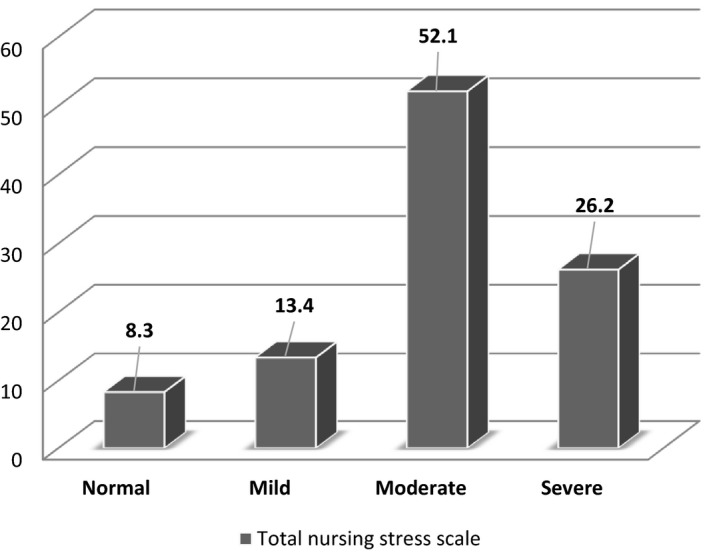
Studied nurses’ distribution in relation to total nursing stress scale (*n* = 374)

Figure 1 indicated that (52.1%) of studied nurses had moderate level of total nursing stress scale. Also, (26.2%) of them had severe level, while (13.4% & 8.3%) of them had mild and normal level, respectively. Figure (1): Studied nurses’ distribution in relation to total nursing stress scale (*n* = 374).

### 3.3 Relation between staff nurses’ demographic characteristics at isolation Hospital and their stress level

Table 3 shown that there was a highly statistically significant relation between total NSS of the studied nurses and their characteristics (*P* ≤ 0.01). Also, there was statistically significant relation with Workplace and educational level at (*P* ≤ 0.05), while there was no statistically significant relation with age, gender and years of experience at (*P* ≥ 0.05). Table (3): Relation between staff nurses’ socio‐demographic characteristics and stress level (*N* = 374).

**TABLE 3:** Relation between staff nurses’ socio‐demographic characteristics and stress level (*N* = 374)

Items		Stress level	test	*P* value
Age	20 to <30	97.50 ± 2.12	*F* test 2.747	.076
	30 to <40	102.08 ± 7.02		
	40–50	96.66 ± 11.93		
Gender	Male	96.50 ± 6.36	T test 1.972	.152
	Female	100.69 ± 7.91		
Marital status	Married	105.57 ± 2.92	T test 5.638	.007**
	Not Married	86.18 ± 4.32		
Have children	Yes	107.25 ± 3.40	T test 7.140	.001**
	No	81.57 ± 8.17		
Work place	Critical care units	100.66 ± 10.44	T test 3.383	.03*
	Ward units	91.06 ± 9.72		
Educational level	Postgraduate	77.83 ± 8.80	*F* test 3.164	.034*
	Bachelor	82.04 ± 10.19		
	Technical	91.13 ± 7.13		
	Diploma	100.13 ± 3.56		
Nursing experience	<10	98.32 ± 2.64	*F* test 2.107	.061
	10 to <20	102.46 ± 9.27		
	≥20	97.21 ± 13.1		

a Significant at *P* < .05. ^**^Highly significant at *P* < .01. Not significant at *P* > .05.*

### 3.4 Relation between staff nurses’ characteristics at isolation Hospital and their stress level

Table 4 showed that there was highly statistically significant relation between total nursing stress scale of the studied nurses and their characteristics as attendance of training courses related to COVID‐19, Fears of infection, Fear of transmission infection for their family, availability of personal protective equipment and nurse to patient ratio at (*P* ≤ 0.01). Also, there was statistically significant relation with surrounding people showed that COVID‐19 is stigma and provide special attention from hospital administration at (P= < 0.05). Table (4) Relation between staff nurses’ characteristics and stress level (*N* = 374).

**TABLE 4:** Relation between staff nurses’ characteristics and stress level (*N* = 374)

Items		Stress level	*T* test	*P* value
Participated in isolation for infectious disease previously	Yes	98.90 ± 12.78	1.461	.120
	No	101.47 ± 9.49		
Training courses related to COVID−19	Yes	82.69 ± 11.83	8.361	.000**
	No	105.44 ± 12.37		
History of psychiatric illness	Yes	96.01 ± 12.56	2.101	.052
	No	90.16 ± 13.07		
Fears of infection	Yes	105.05 ± 12.52	8.412	.000**
	No	80.57 ± 12.95		
Fear of transmission infection for your family	Yes	108.17 ± 11.20	8.500	.000**
	No	81.08 ± 12.51		
Availability of Personal protective equipment	Yes	80.81 ± 12.82	7.236	.001**
	No	104 ± 11.38		
Surrounding people showed that COVID−19 is stigma	Yes	103.71 ± 13.07	3.934	.018*
	No	91.72 ± 12.33		
Provide special attention from hospital administration	Yes	95.3 ± 16.12	3.287	.035*
	No	104.31 ± 14.73		
Nurse to patient ratio	1:1	81.45 ± 13.35	*F* test 8.316	.000**
	1:2	90.44 ± 16.92		
	1:3	98 ± 4.24		
	> 1:3	108.75 ± 14.56		

a Significant at *P* < .05. ^**^Highly significant at *P* < .01. Not significant at *P* > .05.*

### 3.5 Multiple Linear regression model

Table 5 indicated that there was significant statistical positive effect from marital status, have children and people showed that COVID‐19 is stigma on nurses’ stress at *P* value <.05. While there was highly positive effect of fears of infection, workplace, fear of transmission infection for family, nurse to patient ratio on nurses’ stress (*P* = .01), there was highly negative effect of training for COVID‐19 and availability of PPE on nurses’ stress at *P* value <.01. Also, there was slight negative effect of educational level and Attention of hospital administration on nurses’ stress at *P* value <.05. Table (5): Multiple Linear regression model.

**TABLE 5:** Multiple linear regression model

	Unstandardized coefficients	standardized coefficients	*T*	*P* value
	*B*	β		
Marital status	0.118	0.169	1.038	.031*
Have children	0.108	0.160	2.801	.041*
Educational level	−0.152	0.144	1.169	.023*
Workplace	0.244	0.217	3.525	.001**
Training for COVID−19	−0.174	0.165	10.520	.000**
Fears of infection	0.179	0.168	10.611	.000**
Fear of transmission infection for family	0.187	0.210	3.525	.001**
Availability of PPE	−0.218	0.207	10.536	.000**
People showed that COVID−19 is stigma	0.115	0.168	2.464	.014*
Attention of hospital administration	−0.076	0.134	2.243	.026*
Nurse to patient ratio	0.162	0.198	3.186	.002**

ANOVA			
Model	d.f.	*F*	*P* value
Regression	11	5.942	.001**

a. Dependent variable: total nursing stress scale.

b. Predictors: (constant) Marital status, Have children, Educational level, Workplace, Training for COVID‐19, Fears of infection, Fear of transmission infection for family, Availability of PPE, People showed that COVID‐19 is stigma, Attention of hospital administration and Nurse to patient ratio.

## 4 DISCUSSION

The rapid spread of COVID‐19 has put huge burden on health systems around the world. The effects on frontline medical practitioners have also been severe. Nurses are one of the groups at greater risk of infection. However, the negative psychological effects of working on the frontline of the pandemic have also been significant (Mo et al., 2020).

According to nursing stress scale, the results of the current study specified that the highest mean score of nursing stress scale was inadequate preparation, work load and lack of staff support, but the lowest mean score belonged to conflict with other nurse and conflict with physician. These results may be due to half of studied nurses worked related >1–3 nurse to patient ratio which increase workload and more than half of them not attended training courses related to COVID‐19 which negatively affect staff support. It is consistent with the study performed by Pappa et al., 2020, who revealed that at least one in five healthcare professionals report symptoms of depression and anxiety. Also, agreement with the study conducted by Hessels et al., 2019 who reported that Outbreak and exposure response creates a substantial workload for nurses and IPs.

In the present study, the mean score of total nursing stress scale was higher than the mean score. Also, revealed that around half of studied nurses had moderate level of total nursing stress scale. Also, slightly more than one quarter of them had severe level. Which may be attributed to three quarters of studied nurses not participated at previous isolation and more than half of them not provide special attention from hospital administration, most of them fear of transmission infection for their family and more than three quarter fear from infection. These results disagree with the study conducted by Kang et al., 2020 who reported that 36.9% had subthreshold mental health disturbances, 34.4% had mild disturbances, 22.4% had moderate disturbances and 6.2% had severe disturbances. But consistence with the study performed by Jiang, 2020 who revealed that medical staff experienced emotional stress during the COVID‐19 outbreak. Also, cohort with the study by Liang et al., 2020 who detected that Several staff were experiencing clinically significant depressive symptoms. And supported with the study conducted by Lai et al., 2019 who reported that 50.4% reported symptoms of depression, 44.6% anxiety, 34.0% insomnia and 71.5% reported distress. Meanwhile, cohort with the study by Weilenmann et al., 2020 who detected that overall symptom levels of anxiety, depression and burnout were elevated.

In this study, regarding linear model, the results showed that the training for COVID‐19, availability of PPE, educational level and attention of hospital administration were negative predictor factors for nurses’ stress, which may be due to knowledge about COVID‐19 is limited, so training programme decreasing stress level. Strengthening specialist training and preparation is the only effective measure to alleviate the psychological pressure of the medical staff (Grace & VanHeuvelen, 2019). These results supported with the study conducted by Chu et al., (2020) who reported that training prevention was predictor factor for nurses’ stress. Also, agree with the study performed by Jeong et al., 2016 who presented that providing inadequate basic supplies during quarantine was a source of frustration and continued to be associated with anxiety and stress. Meanwhile, two studies also found that those who believed there to be a shortage of personal protective equipment also had high stress (Lu et al., 2020 and Chung & Yeung, 2020).

Also, marital status, have children, people showed that COVID‐19 is stigma, effect of fears of infection, workplace, fear of transmission infection for family, nurse to patient ratio were positive predictors for nurses’ stress. Nurses fear from infection (85%), having children (44.4%) and working in critical units (29.4%) suffered from high stress. Nurses worry about the health of their family members and fear that will lose one of their family. When various roles are in conflict, certain psychological burden will be present. These results consistent with the study done by Xiao et al., 2020 who detected that levels of social support were negatively associated with the degree of anxiety and stress. Also, supported with Huang et al., 2019 who detected that long working time per week increased stress, which may be correlated with the fear of infection and excessive physical consumption. And agreement with the study conducted by Lai et al., 2020 who revealed that burden care of patients with COVID‐19 was associated with a higher risk of symptoms of depression (OR, 1.52; 95% CI, 1.11–2.09; *P* =  .01), anxiety (OR, 1.57; 95% CI, 1.22–2.02; *P* <  .001). Meanwhile, supported with Maunder, 2004 who reported that having children and stigmatization were relevant factors related to healthcare workers’ stress.

## 5 CONCLUSIONS

The results showed that half of studied nurses at isolation hospitals had moderate level of stress. Also, more than one quarter of them had severe level. Training for COVID‐19, availability of PPE, educational level and attention of hospital administration were negative predictor factors for nurses’ stress. Have children, people showed that COVID‐19 is stigma, effect of fears of infection, workplace, fear of transmission infection for family, nurse to patient ratio were positive predictors for nurses’ stress.

## 6 CONFLICTING INTERESTS

The author(s) declared no potential conflicts of interest with respect to the research, authorship and/or publication of this article.

## AUTHORS’ CONTRIBUTION

All authors contributed to the study conception, design, data collection, data analysis and wrote all parts of the manuscript. All authors read, commented on and approved the final manuscript.

## Data Availability

The data are not publicly available due to the restrictions, for example they contain information that could compromise the privacy of research participants.
